# Investigation of the Effect of Exposure to Liquid Chemicals on the Strength Performance of 3D-Printed Parts from Different Filament Types

**DOI:** 10.3390/polym17121637

**Published:** 2025-06-12

**Authors:** Arslan Kaptan

**Affiliations:** Motor Vehicles and Transportation Technologies Department, Sivas Technical Sciences Vocational School, Sivas Cumhuriyet University, 58140 Sivas, Turkey; akaptan@cumhuriyet.edu.tr; Tel.: +90-3464875472

**Keywords:** additive manufacturing, chemical resistance, 3D printing filament materials, tensile strength, impact resistance, material selection

## Abstract

Additive manufacturing (AM), particularly fused deposition modeling (FDM) 3D printing, has emerged as a versatile and accessible technology for prototyping and functional part production across a wide range of industrial applications. One of the critical performance-limiting factors in AM is the chemical resistance of thermoplastic materials, which directly influences their structural integrity, durability, and suitability in chemically aggressive environments. This study systematically investigates the chemical resistance of eight different widely utilized FDM filaments—acrylonitrile butadiene styrene (ABS), acrylonitrile styrene acrylate (ASA), polyamide (PA, Nylon), polycarbonate (PC), polyethylene terephthalate glycol (PETG), polylactic acid (PLA), polypropylene (PP), and polyvinyl butyral (PVB)—by examining their tensile strength and impact resistance after immersion in representative chemical agents: distilled water, ethanol (99.5%), isopropyl alcohol (75% and 99%), acetic acid (8%), hydrochloric acid (37%), hydrogen peroxide (30%), and acetone (99.5%). Quantitative mechanical testing was conducted in accordance with ASTM D638 and ASTM D256 standards, and statistical variability was accounted for using triplicate measurements with standard deviation analysis. The results reveal that PP exhibits the highest chemical resilience, retaining over 97% of its mechanical properties even after 7 days of immersion in aggressive solvents like acetone. PETG and ASA also demonstrated quite successful stability (>90% retention) in mildly corrosive environments such as alcohols and weak acids. In contrast, PLA, due to its low crystallinity and polar ester backbone, and PVB, due to its high amorphous content, showed substantial degradation: tensile strength losses exceeding 70% and impact resistance dropping below 20% in acetone. Moderate resistance was observed in ABS and PC, which maintained structural properties in neutral or weakly reactive conditions but suffered mechanical deterioration (>50% loss) in solvent-rich media. A strong correlation (r > 0.95) between tensile and impact strength reduction was found for most materials, indicating that chemical attack affects both static and dynamic mechanical performance uniformly. The findings of this study provide a robust framework for selecting appropriate 3D printing materials in applications exposed to solvents, acids, or oxidizing agents. PP is recommended for harsh chemical environments; PETG and ASA are suitable for moderate exposure scenarios, whereas PLA and PVB should be limited to low-risk, esthetic, or disposable applications.

## 1. Introduction

Additive manufacturing (AM), particularly fused deposition modeling (FDM)-based three-dimensional (3D) printing, has revolutionized the fabrication of complex, customized components across diverse sectors including aerospace, automotive, biomedical, and consumer products [1,2,3]. The primary advantage of AM lies in its ability to produce intricate geometries with minimal material waste, reduced lead times, and enhanced design flexibility [4,5]. Over the past decade, the range of materials available for 3D printing has expanded significantly—from conventional thermoplastics such as PLA and ABS to more advanced polymers like PA, PC, and PETG, as well as composite- and fiber-reinforced variants [6,7,8,9,10].

Materials such as PLA, although biodegradable and cost-effective, exhibit low resistance to polar solvents and acidic media due to their polar ester backbone and relatively low crystallinity, which facilitate solvent-induced diffusion and hydrolytic degradation [11,12]. Conversely, PP and PETG offer enhanced chemical durability owing to their non-polar or semi-crystalline molecular structures [13,14,15,16,17,18]. Despite these known trends, the existing literature lacks a systematic, comparative evaluation of how such materials degrade mechanically over time in realistic chemical environments.

In practice, components manufactured via FDM are increasingly deployed in chemically active domains—such as laboratory fixtures, medical devices, fluid handling systems, and environmental sensors—where sustained contact with reactive agents can compromise part integrity [19,20,21,22,23,24]. Therefore, beyond assessing baseline mechanical strength (e.g., tensile and impact resistance), it is essential to quantify how these properties evolve under chemical stress and how different polymer chemistries respond to such conditions.

Although several studies have reported short-term chemical compatibility tests, comprehensive long-duration immersion studies under multiple chemical categories (e.g., solvents, acids, oxidants) remain scarce [25,26,27]. Furthermore, many of these investigations fail to correlate degradation patterns with material structure or provide comparative insights necessary for informed material selection in industrial applications. Studies suggest that the incorporation of filler phases, polymer blending, or surface functionalization may enhance resistance; however, foundational reference data for the unmodified commercial-grade filaments is still needed to validate such improvements [28,29,30,31,32].

In contrast, materials like PETG and PP have shown better resistance to solvents and acids, making them suitable for more demanding applications [33,34,35]. However, the balance between mechanical strength and chemical resistance remains a critical area of development [36,37]. Studies indicate that combining multiple polymers or reinforcing base polymers with fibers can significantly enhance both chemical stability and structural integrity, thus expanding their applications in harsh environments [38,39,40,41,42,43,44,45,46,47].

While the mechanical properties of these materials have been widely investigated, their chemical resistance remains an underexplored yet critically important aspect—particularly in applications involving direct exposure to solvents, acids, oxidizers, or alcohols. This study addresses this critical gap by experimentally evaluating the chemical resistance of eight commercial FDM filaments—ABS, ASA, PA (Nylon), PC, PETG, PLA, PP, and PVB—through mechanical performance testing after immersion in a representative range of industrial chemicals: distilled water, ethanol (99.5%), isopropyl alcohol (75% and 99%), acetic acid (8%), hydrochloric acid (37%), hydrogen peroxide (30%), and acetone (99.5%). The specimens were exposed for three durations (1 h, 24 h, and 7 days) and tested according to ASTM D638 [48] and ASTM D256 [49] standards. In this study, tensile and impact tests were preferred to be performed in determining the strength performances. The reason for this is that, on the one hand, the critical values for the samples are considered to be tensile and impact tests. Since higher values will be obtained in the compressive strength tests, they were not evaluated as critical performance values for the filaments. On the other hand, due to the variety of chemicals used (eight different liquid chemicals) and the abundance of filament types (eight different filament types), the experimental studies were not included because they were too large to fit into this study. This work aims to establish a structure–property–performance correlation, quantify degradation trends, and provide a decision-making framework for selecting FDM materials in chemically demanding environments.

## 2. Materials and Methods

### 2.1. Material

This study investigates the chemical resistance of eight commercially available thermoplastic filaments widely used in FDM: acrylonitrile butadiene styrene (ABS), acrylonitrile styrene acrylate (ASA), polyamide (PA, Nylon), polycarbonate (PC), polyethylene terephthalate glycol (PETG), polylactic acid (PLA), polypropylene (PP), and polyvinyl butyral (PVB) (Sigma-Aldrich and Merc, St Louis, MO, USA). These materials were selected to represent a broad spectrum of mechanical performance, thermal behavior, and chemical reactivity, thereby providing a comprehensive basis for comparative evaluation under chemical stress.

The rationale behind material selection is twofold. Firstly, from an application standpoint, the chosen filaments span from basic prototyping plastics (e.g., ABS, PLA) to high-performance engineering-grade polymers (e.g., PA, PC, PP). Secondly, in terms of molecular architecture, they exhibit significant diversity—from amorphous to semi-crystalline morphologies, from hydrophilic to hydrophobic backbones, and from polar to non-polar functionalities—making them ideal candidates for correlating structure–property–performance relationships under chemical exposure. All filaments were procured from leading manufacturers with standardized documentation for consistency.

All filaments were stored in a desiccated cabinet at 20 ± 2 °C and humidity below 15% RH to minimize moisture absorption prior to testing. Each spool was inspected for consistency in diameter and roundness (tolerance ±0.03 mm) using a digital micrometer to ensure uniform extrusion during printing. Table 1 shows the short names of filament materials, their trade names, manufacturers, and countries of origin. A summary of the key mechanical characteristics and general application areas is presented in Table 2, providing foundational context for analyzing how different chemical environments affect material behavior.

### 2.2. Chemicals

Ethanol (99.5%), isopropyl alcohol (75% and 99%), acetic acid (8%), hydrochloric acid (37%), hydrogen peroxide (30%), and acetone (99.5%) were obtained from Sigma-Aldrich (St Louis, MO, USA). All other chemicals of analytical grade were purchased from Merck (St Louis, MO, USA).

To evaluate the chemical durability of the selected 3D printing materials under realistic and application-relevant conditions, a diverse set of seven chemical agents was selected. The chemicals were chosen based on their frequency of use in industrial, biomedical, and laboratory environments, as well as their ability to represent a spectrum of chemical reactivity. All chemical immersion tests were performed using analytical grade reagents in sealed borosilicate glass containers at room temperature (22 ± 2 °C). For each test condition, 500 mL of chemical solution was used per specimen to ensure complete submersion. The immersion durations were set at 1 h, 24 h, and 7 days, simulating short-term contact, daily operational exposure, and prolonged chemical stress, respectively. Upon completion of each exposure period, the samples were rinsed with distilled water, air-dried for 24 h, and then subjected to mechanical testing. All procedures were conducted under a fume hood with appropriate personal protective equipment, in accordance with laboratory safety protocols. Table 3 summarizes the selected chemicals, along with their formulas, code names of chemicals (R1, R2, …, R8) used throughout this article, classifications, and typical areas of application, and contextualizes their industrial importance and material interaction mechanisms.

### 2.3. Specimen Preparation Experimental Procedure

**Figure 1:**
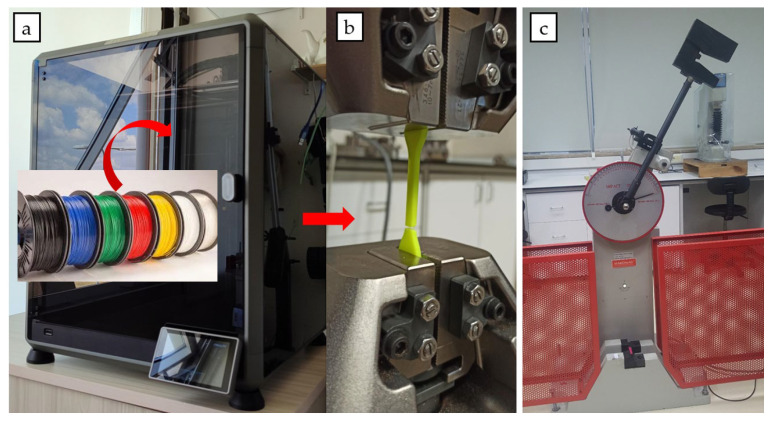
Printing and testing of experiment sample: (**a**) Creality K1 max 3D printer, (**b**) tensile test setup, (**c**) impact test setup.

All test specimens were fabricated using a K1 Max 3D printer (Creality, Shenzhen, China) Fused Filament Fabrication (FFF) and filaments (Figure 1a), operating within a controlled ambient chamber at 22 ± 2 °C. To ensure experimental reproducibility and minimize print-to-print variability, all process parameters were strictly standardized across materials. Test geometries were modeled and sliced in accordance with ASTM D638 [48] Type V for tensile testing (Figure 1b) and ASTM D256 [49] for Izod impact testing (Figure 1c). For impact testing, a standardized notch was introduced, perpendicular to the loading direction, using a precision notch cutter. After fabrication, samples were subjected to static chemical immersion tests in sealed borosilicate glass vessels containing 100 mL of each chemical agent at ambient temperature (22 ± 2 °C). The exposure durations were defined as short-term (1 h), mid-term (24 h), and long-term (7 days). Immediately following chemical exposure, all samples were rinsed with distilled water, then air-dried for 24 h in a dust-free environment before mechanical testing. Tensile strength testing was carried out using a Instron 3367, Autograph AGS-X universal testing machine (Shimadzu, Kyoto, Japan) with a crosshead speed of 5 mm/min and a gauge length of 50 mm. Impact resistance was evaluated using a HIT 25P Charpy impact tester (Zwick/Roell, Ulm, Germany) under notched configuration. Each test condition (material × chemical × duration) was replicated three times (*n* = 3) to ensure statistical significance and mean ± standard deviation (SD) values were recorded. The parameters selected during the production process of all samples are shown in Table 4.

**Figure 2:**
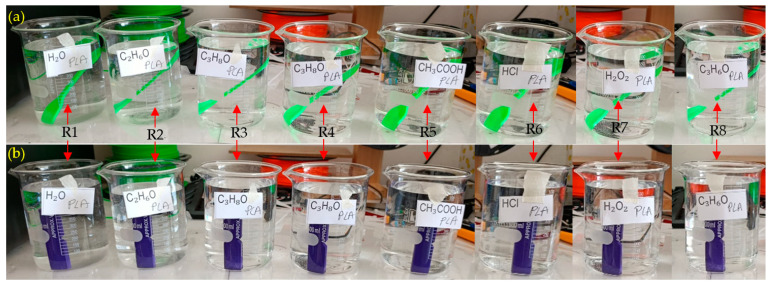
Exposure of PLA to different liquid chemicals, R1: water, R2: ethanol (99.5%), R3: IPA (75%), R4: IPA (99%), R5: acetic acid (8%), R6: hydrochloric acid (37%), R7: hydrogen peroxide (30%), R8: acetone (99.5%): (**a**) tensile samples, (**b**) impact samples.

Figure 2 shows the exposure of tensile (Figure 2a) and impact test samples (Figure 2b) printed with PLA, one of the filaments used in the experiment, to liquid chemicals. All test samples were in contact with the chemical, and this process was repeated three times for each sample.

**Figure 3:**
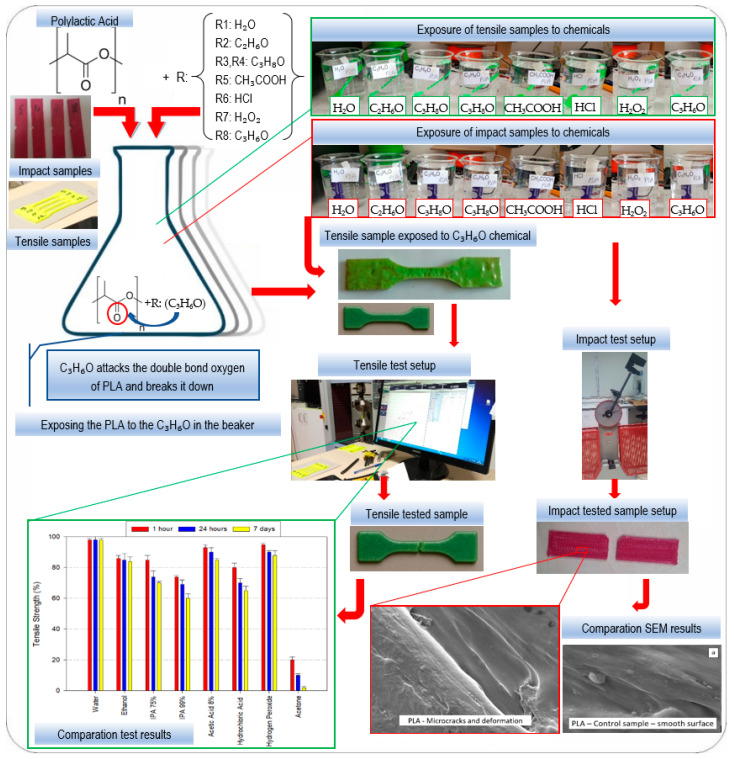
Experimental workflow for chemical exposure, mechanical testing, and SEM results.

The full experimental workflow, including fabrication, immersion, and post-processing, is summarized schematically in Figure 3. The findings presented in this study are specific to static immersion at room temperature. Future studies will incorporate elevated thermal exposure, dynamic agitation, and cyclical loading conditions to better emulate real-world service environments. In this study, surface roughness (Ra) measurements were not conducted. This decision is based on the fact that chemical degradation causes significant morphological changes on the sample surfaces, such as microcracks, delamination, erosion, and void formation, as observed in scanning electron microscopy (SEM) analysis, as discussed in Section 3.5. These irregularities led to non-uniform and inconsistent surface topographies, making it impractical to obtain reliable and repeatable Ra values using conventional contact-based profilometry. As a result, any roughness data derived from such degraded surfaces would not be representative of true topological characteristics but rather reflect localized damage or chemical artifacts. Instead, morphological changes were qualitatively assessed using high-resolution SEM, which provided a more accurate visualization of surface integrity and degradation mechanisms.

## 3. Results

### 3.1. Tensile Strength and Izod Impact Test

To establish a quantitative baseline for evaluating the mechanical degradation resulting from chemical exposure, unconditioned reference specimens of each 3D printing filament were first tested under standard laboratory conditions. Tensile strength was measured according to the ASTM D638 [48] standard using a universal testing machine, while Izod impact resistance was determined in accordance with ASTM D256 [49], employing a notched configuration to simulate impact failure mechanisms. Each mechanical test was conducted on three replicate specimens (*n* = 3) to ensure statistical robustness. The results are expressed as mean values ± SD, enabling error quantification and facilitating reliable comparison with chemically exposed specimens in subsequent analyses. These baseline measurements represent the intrinsic mechanical properties of the materials in their as-printed state and are crucial for assessing percentage reductions in strength performance following chemical immersion. Specifically, they serve as the control dataset for computing the relative retention of mechanical properties after 1 h, 24 h, and 7-day exposure periods. Table 5 presents the average tensile strength (in MPa) and Izod impact resistance (in J/m) for all eight filament types prior to chemical treatment. This foundational dataset forms the basis for the material-specific degradation profiles discussed in the following subsections.

### 3.2. Filaments Used in Chemical Resistance Analysis Tests Applied

#### 3.2.1. Tests Performed on Acrylonitrile Butadiene Styrene (ABS) Filament

**Figure 4:**
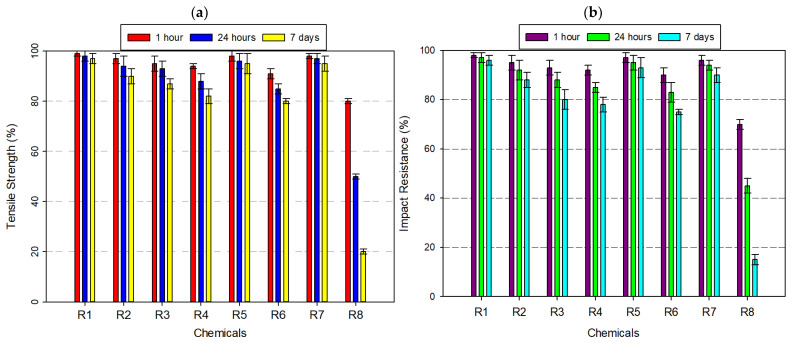
Graph of the effects of different chemicals on ABS filament over time: (**a**) tensile strength; (**b**) impact resistance.

ABS exhibited moderate resistance to chemical degradation, with its mechanical performance varying significantly depending on the chemical type and exposure duration (Figure 4a,b). As a rubber-toughened amorphous polymer, ABS benefits from the energy-absorbing capacity of its butadiene domains and the rigidity of styrene-acrylonitrile phases, which together determine its chemical stability profile. Under neutral conditions (distilled water), ABS retained approximately 99% of its original tensile strength and 98% of its Izod impact resistance, even after 7 days, indicating quite good resistance to hydrolytic effects due to its non-polar backbone and limited water absorption. In mildly aggressive media, such as 8% acetic acid and 30% hydrogen peroxide, the mechanical performance showed only slight degradation—tensile strength dropped to ~95%, and impact resistance ranged between 90% and 93%. However, alcohol-based solvents—particularly IPA at high concentration—initiated a gradual deterioration. After 7 days of exposure to 99% IPA, tensile strength declined to 82% and impact resistance fell to 78%. In strong acidic environments such as 37% hydrochloric acid, ABS exhibited further vulnerability. After prolonged exposure, the tensile strength and impact resistance dropped to ~80% and 75%, respectively. The most severe deterioration occurred under acetone exposure—a solvent known for its strong affinity to styrenic polymers. After 7 days, ABS specimens retained only ~20% of their tensile strength and ~15% of their impact resistance.

#### 3.2.2. Tests Performed on Acrylonitrile Styrene Acrylate (ASA) Filament

**Figure 5:**
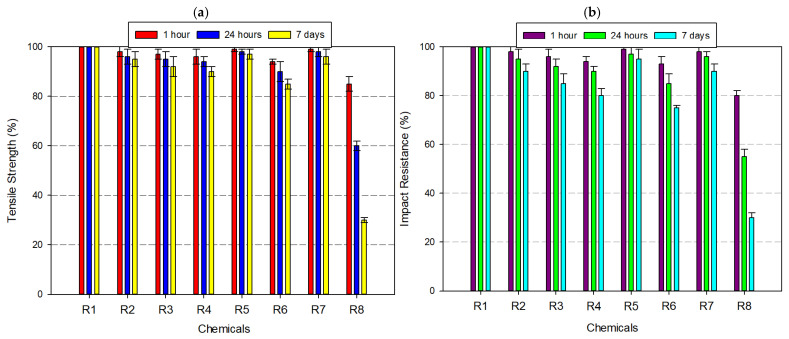
Graph of the effects of different chemicals on ASA filament over time: (**a**) tensile strength; (**b**) impact resistance.

ASA exhibited high chemical stability across a broad spectrum of exposure conditions, maintaining substantial tensile and impact strength in neutral, oxidizing, and weakly acidic environments (Figure 5a,b). Engineered as a weather-resistant alternative to ABS, ASA incorporates an acrylate-modified styrenic backbone stabilized with UV-resistant agents, contributing to its robust performance in outdoor and chemically moderate applications. After 7 days of immersion in distilled water, ASA specimens showed no measurable loss in mechanical integrity, retaining 100% of both tensile and impact strength. Similarly, in 8% acetic acid and 30% hydrogen peroxide, retention levels remained above 95%, highlighting the material’s oxidative and hydrolytic stability under mildly reactive conditions. When exposed to ethanol (99.5%), ASA experienced a slight decline, with tensile strength reduced to ~95% and impact resistance to ~90%. More pronounced degradation occurred under isopropyl alcohol, especially at 99% concentration, where tensile and impact properties decreased to approximately 90% and 80%, respectively. In 37% hydrochloric acid, ASA’s mechanical properties declined further—tensile strength fell to 85% and impact resistance to 75% after 7 days. The most significant degradation was observed under acetone exposure, a powerful organic solvent known for solvating styrenic phases. Within just 24 h, tensile strength dropped to 60% and by day 7, fell to 30%, with a corresponding impact resistance decline to 30%. Despite its vulnerability to aggressive solvents like acetone, ASA remains a highly favorable candidate for moist, oxidizing, and moderately acidic environments—particularly in UV-exposed outdoor settings. However, applications involving prolonged solvent exposure (e.g., alcohol-based disinfectants or industrial degreasers) require material substitution or protective coating strategies to prevent mechanical failure.

#### 3.2.3. Tests Performed on Polyamide (PA) Filament

**Figure 6:**
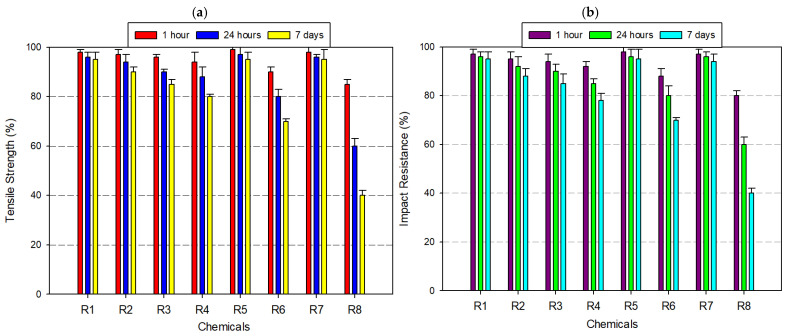
Graph of the effects of different chemicals on PA filament over time: (**a**) tensile strength; (**b**) impact resistance.

PA, commonly known as nylon, displayed a chemically selective resistance profile, characterized by stability in neutral and mildly reactive media and notable vulnerability in strongly polar or acidic environments (Figure 6a,b). After 7 days of immersion in distilled water, 8% acetic acid, and 30% hydrogen peroxide, PA retained approximately 95% of its initial tensile and impact strength, confirming its suitability for moisture-rich and oxidative conditions. Under ethanol (99.5%) exposure, tensile and impact resistance dropped to ~90%, while 99% IPA caused further degradation, with mechanical retention reduced to 80–85%. These findings suggest that solvent polarity, not just concentration, plays a central role in weakening the amorphous matrix via penetrative diffusion and molecular swelling. In the presence of 37% hydrochloric acid, PA underwent more severe degradation. Tensile and impact strength declined to approximately 70%. Acetone exposure yielded the most dramatic deterioration. Within just 24 h, mechanical properties fell to ~60%, and by day 7, both tensile and impact resistance values declined to ~40%. These observations affirm that PA is a high-performance material for wet, mildly acidic, or oxidizing conditions, particularly in engineering applications requiring flexibility and impact resistance (e.g., gears, fluid connectors, bushings). However, its deployment in polar solvent-rich or strongly acidic environments should be approached with caution. In such cases, surface coatings, polymer blends, or post-treatment stabilizers may be necessary to mitigate structural degradation and maintain long-term functionality.

#### 3.2.4. Tests Performed on Polycarbonate (PC) Filament

**Figure 7:**
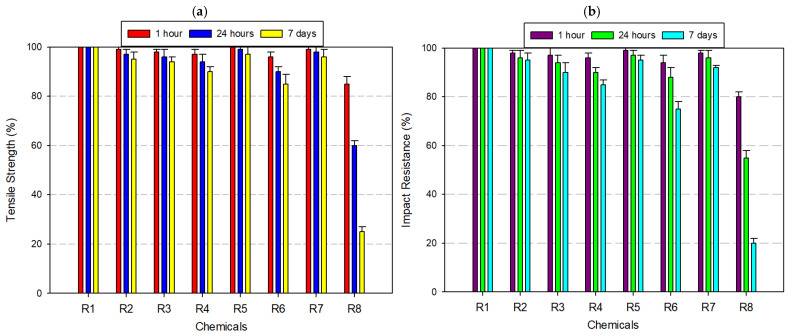
Graph of the effects of different chemicals on PC filament over time: (**a**) tensile strength; (**b**) impact resistance.

PC exhibited strong resistance to moisture, weak acids, and oxidizing agents, maintaining full mechanical integrity in aqueous and mildly reactive environments (Figure 7a,b). Its amorphous structure, low water uptake, and excellent optical clarity, making PC a preferred choice for mechanical parts and enclosures subjected to humid operating conditions. After 7 days of immersion in distilled water, 8% acetic acid, and 30% hydrogen peroxide, PC preserved ≥95% of its original tensile and impact strength. Exposure to alcohols such as ethanol (99.5%) and IPA caused moderate degradation, with tensile strength and impact resistance dropping to ~90% and 85%, respectively. Upon exposure to 37% hydrochloric acid, mechanical properties further declined, with tensile strength dropping to ~85%, and impact resistance to ~75%. The most critical deterioration occurred under acetone exposure, where PC’s amorphous morphology became a liability. After only 24 h, tensile strength dropped to 60% and impact resistance fell to ~40%. By day 7, these values had further declined to 25% (tensile) and 20% (impact). Despite this, PC remains an ideal candidate for high-stress applications in medical, optical, and mechanical fields, where limited solvent exposure is expected. However, in solvent-prone or acid-rich environments, the use of protective coatings, multi-layer designs, or alternative chemically resistant materials is strongly recommended to maintain long-term performance.

#### 3.2.5. Tests Performed on Polyethylene Terephthalate Glycol (PETG) Filament

**Figure 8:**
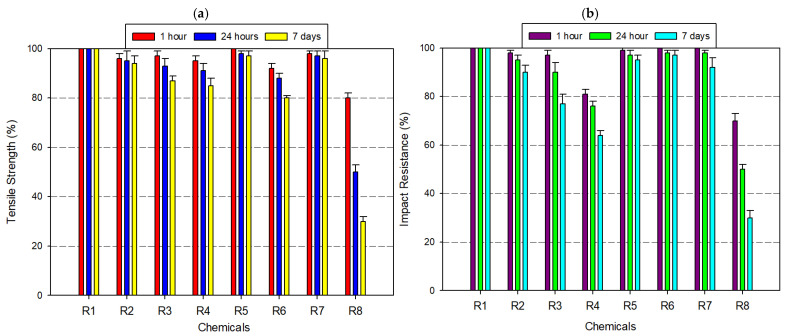
Graph of the effects of different chemicals on PETG filament over time: (**a**) tensile strength; (**b**) impact resistance.

PETG exhibited excellent chemical resistance under a wide range of exposure conditions, particularly in neutral, mildly acidic, and oxidizing environments. The material’s semi-crystalline architecture and glycol modification contribute to its hydrophobicity and resistance to hydrolytic degradation, making it an optimal candidate for applications involving moisture, low-pH agents, or oxidants (Figure 8a,b). After 7 days of immersion, PETG retained over 97% of its tensile strength and 92–97% of its impact resistance in distilled water, 8% acetic acid, and 30% hydrogen peroxide. In ethanol (99.5%), decreases were also observed—tensile strength decreased to approximately 94%, and impact resistance to ~90%. However, with 99% isopropyl alcohol, PETG demonstrated increased sensitivity; tensile and impact values declined to ~85% and ~64%, respectively. Exposure to 37% hydrochloric acid induced further degradation—tensile strength fell to ~80%, and impact resistance to ~72%. The most pronounced deterioration occurred in acetone, a potent solvent for glycol-modified polyesters. Within 24 h, tensile strength dropped to ~50%, and by day 7, it further declined to ~30%, with impact resistance following a similar trend. In summary, PETG offers high mechanical stability and chemical compatibility in aqueous, oxidizing, and mildly acidic environments, making it highly suitable for food packaging, medical containers, transparent housings, and industrial prototyping. However, its limited resistance to ketones and high-polarity organic solvents such as acetone and high-purity IPA demands careful material selection or protective strategies in solvent-rich environments.

#### 3.2.6. Tests Performed on Polylactic Acid (PLA) Filament

**Figure 9:**
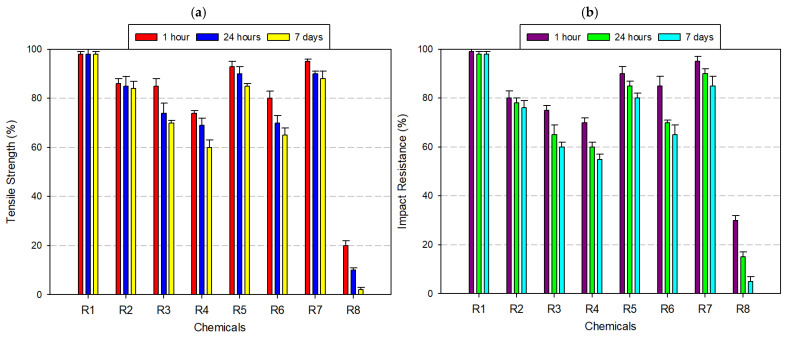
Graph of the effects of different chemicals on PLA filament over time: (**a**) tensile strength; (**b**) impact resistance.

PLA, a widely used biodegradable thermoplastic, exhibited a highly selective chemical resistance profile, with stable performance in benign aqueous conditions and rapid degradation under exposure to strong acids and polar organic solvents (Figure 9a,b). After 7 days of immersion, PLA retained nearly 100% of its tensile and impact strength in distilled water and maintained approximately 85% of both properties in 8% acetic acid and 30% hydrogen peroxide. The semi-crystalline domains in PLA restrict chain mobility and create a densely packed phase, limiting solvent access to the amorphous regions where degradation typically initiates.

Similar findings have been reported in previous studies. For instance, Sodergard and Stolt (2002) explain that the crystallinity in PLA significantly influences its solvent resistance by limiting polymer chain accessibility to small molecules [50]. Likewise, Rasal et al. (2010) emphasize that the hydrophobic character of PLA reduces its permeability to polar fluids such as water and ethanol (99.5%), contributing to its relative stability in mild environments [51]. These structural features collectively explain PLA’s better resistance in low-reactivity conditions, as observed in our mechanical and morphological evaluations. However, the material’s chemical vulnerability sharply increased in ethanol (99.5%) and isopropyl alcohol. In ethanol (99.5%), mechanical retention declined to ~85% (tensile) and ~76% (impact) after 7 days. More aggressive degradation was observed with 99% isopropyl alcohol, where tensile strength dropped to ~60–70% and impact resistance to ~55–60%. In 37% hydrochloric acid, PLA underwent accelerated degradation, with both tensile and impact strength declining to ~65%. The most catastrophic degradation occurred in acetone, a ketone solvent with high affinity for PLA’s ester-functionalized backbone. Within just 1 h, tensile strength plummeted to ~20%, and by day 7, both tensile and impact resistance approached 0% and 5%, respectively. In summary, while PLA is an eco-friendly, low-cost, and accessible filament ideal for esthetic prototyping, biomedical scaffolds, and non-reactive enclosures, its use should be strictly limited to low-solvent, low-acidity, and non-polar environments. For applications requiring higher chemical resilience, the integration of blending techniques, surface coatings, or barrier treatments is recommended to extend PLA’s utility without compromising structural performance.

#### 3.2.7. Tests Performed on Polypropylene (PP) Filament

**Figure 10:**
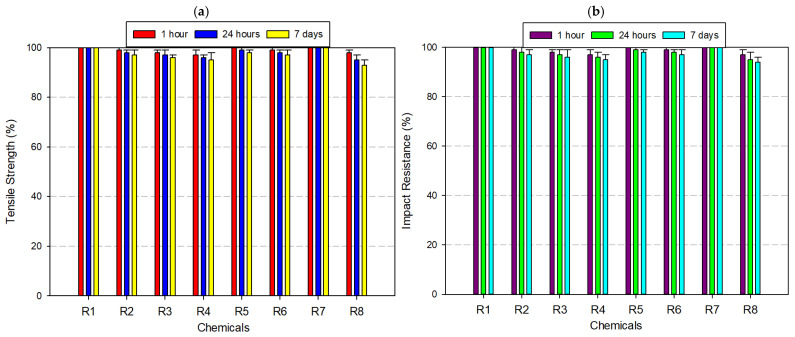
Graph of the effects of different chemicals on PP filament over time: (**a**) tensile strength; (**b**) impact resistance.

PP demonstrated superior chemical resistance across all tested environments, emerging as the most chemically stable material among the evaluated 3D printing filaments. Its semi-crystalline, non-polar, saturated hydrocarbon structure imparts exceptional resistance to molecular diffusion, hydrolysis, and oxidative degradation, positioning PP as a premier candidate for long-term deployment in chemically aggressive conditions (Figure 10a,b). Following 7 days of immersion, PP retained ~100% of its original tensile and impact strength in distilled water and 30% hydrogen peroxide, validating its hydrophobic character and oxidation-resistant backbone. In ethanol (99.5%), 8% acetic acid, and even 37% hydrochloric acid, only minor reductions were observed (tensile and impact strength ≥97–98%), indicating remarkable tolerance even under prolonged acidic or alcoholic exposure. Notably, IPA at both 75% and 99% concentrations caused negligible degradation, with mechanical retention remaining above 95%. The only measurable decline occurred under acetone exposure, where both tensile and impact strength fell slightly to ~93–94% after 7 days. These features make PP exceptionally well-suited for industrial fluid transport systems, chemical-resistant packaging, biomedical enclosures, and exterior mechanical components exposed to a variety of corrosive substances. In summary, PP stands out as a benchmark filament for chemically demanding environments in the FDM domain. While prolonged exposure to strong organic solvents such as acetone may lead to superficial aging effects, its core mechanical integrity remains intact, affirming its status as one of the most chemically inert and application-flexible thermoplastics available to additive manufacturing practitioners.

#### 3.2.8. Tests Performed on Polyvinyl Butyral (PVB) Filament

**Figure 11:**
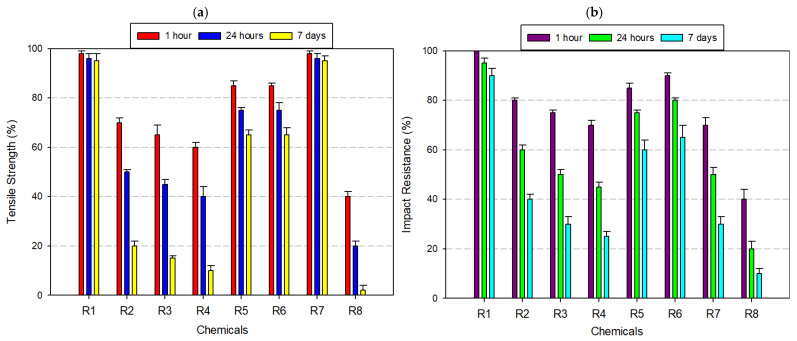
Graph of the effects of different chemicals on PVB filament over time: (**a**) tensile strength; (**b**) impact resistance.

PVB exhibited the lowest chemical resistance among the tested FDM materials, displaying rapid and irreversible mechanical degradation upon exposure to polar solvents and aggressive organic media. While its initial performance in distilled water was acceptable—retaining ~90% of impact resistance after 7 days—its stability in more reactive environments declined markedly (Figure 11a,b). These characteristics render PVB especially sensitive to alcohol-based solvents, such as ethanol (99.5%) and isopropyl alcohol. After 7 days of immersion in 99% isopropyl alcohol, tensile strength decreased by >40%, and impact resistance fell by ~75%. The most severe degradation was observed under acetone exposure. Within 24 h, mechanical properties deteriorated significantly, and by day 7, both tensile and impact strength dropped below 10%. Even in mildly reactive environments such as 8% acetic acid, 37% hydrochloric acid, and 30% hydrogen peroxide, PVB experienced mechanical declines of 25–40%, reflecting a general instability of its network in the presence of acidic or oxidative agents. Although PVB offers optical clarity, flexibility, and adhesive functionality, especially in laminated glass, protective coatings, and photovoltaic modules, its poor chemical durability restricts its use in load-bearing, outdoor, or chemically exposed environments. To extend its functional lifetime, protective barrier layers, coextrusion strategies, or nanofiller reinforcement may be necessary to mitigate degradation and enhance mechanical robustness under solvent stress.

### 3.3. Comparison of Chemically Exposed Filaments

#### 3.3.1. Tensile Strength Comparison

**Figure 12:**
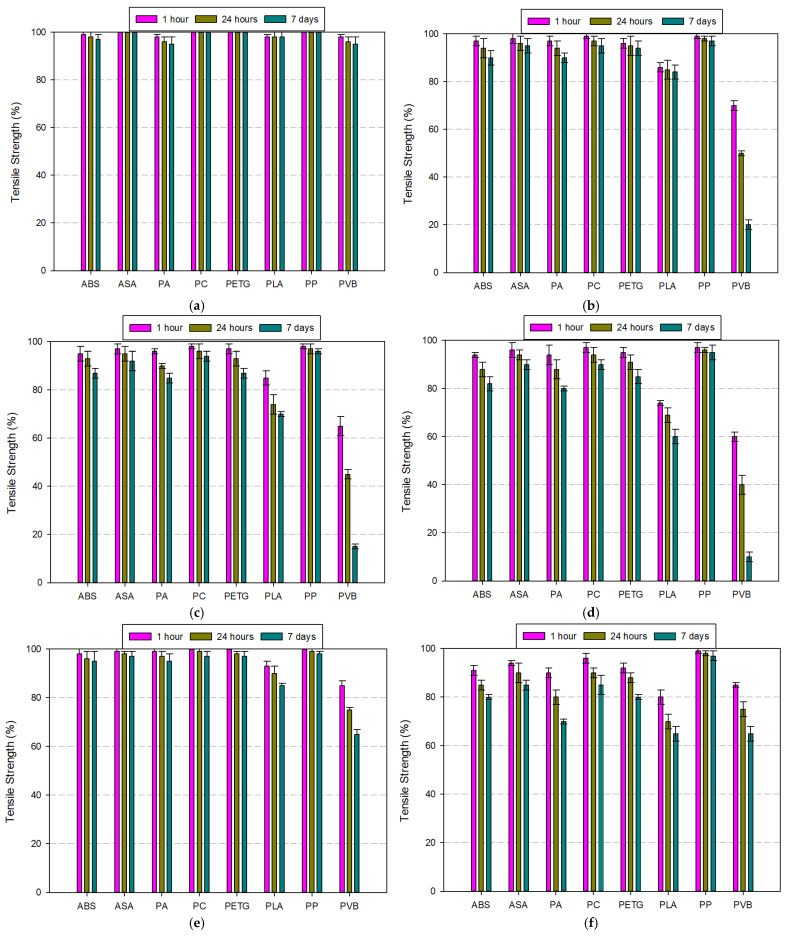

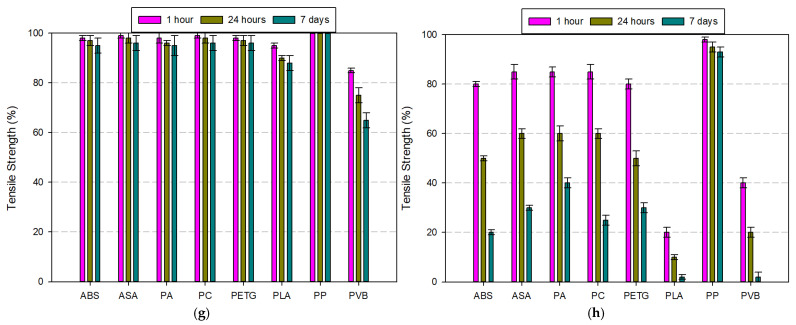
Comparison of tensile strengths of filament materials according to the fluids used: (**a**) water, (**b**) ethanol (99.5%), (**c**) IPA (75%), (**d**) IPA (99%), (**e**) acetic acid (8%), (**f**) hydrochloric acid (37%), (**g**) hydrogen peroxide (30%), (**h**) acetone (99.5%).

The tensile strength of eight different filament materials (ABS, ASA, PA, PC, PETG, PLA, PP, and PVB) was evaluated after immersion in various chemical environments for 1 h, 24 h, and 7 days. Figure 12a–h show the normalized tensile strength retention (%) under different chemical exposures. The results reveal significant material-dependent differences in mechanical degradation behavior over time. Acetone (99.5%) caused the most dramatic strength degradation across all materials, particularly in PLA and PVB, which retained less than 10% of their initial strength after 7 days. PETG also showed a considerable decline, while materials like ASA, PA, and PC maintained relatively better resistance. PP was the most stable filament under this solvent, with minimal reduction. Water exposure, in contrast, showed the least detrimental effect on all filaments. Tensile strength retention remained above 95% for most materials after 7 days, indicating negligible hydrolytic degradation. Only PVB exhibited a moderate decline, suggesting slight water sensitivity. Ethanol (99.5%) and IPA (75% and 99%) had selective impacts. PLA and PVB were again the most affected, with PVB dropping below 20% strength in all alcohol-based media. In contrast, ABS, ASA, PA, and PC showed high retention levels (>90%), indicating good solvent resistance. Acetic acid (8%) resulted in mild degradation for most materials. PLA and especially PVB displayed more pronounced loss over time, whereas PC, PETG, and PP maintained strength above 90%. Hydrochloric acid (37%) exposure significantly affected PVB, with tensile strength falling to approximately 20% after 7 days. PLA and PETG also showed a moderate decline. Meanwhile, ABS, ASA, and PA were notably more stable. Hydrogen peroxide (30%), being a strong oxidizing agent, had a marked effect on PVB and PLA. PVB fell to around 65% and PLA to 85% after prolonged exposure. PETG exhibited a slight decline, while ABS, ASA, and PP remained stable. Overall, PVB and PLA were the most chemically vulnerable filaments, consistently showing significant tensile strength degradation across all solvents and time intervals. In contrast, PP, ASA, and PC demonstrated robust chemical resistance, making them suitable candidates for applications involving harsh chemical environments. These findings emphasize the critical importance of material selection based on anticipated chemical exposure, especially for structural applications, where mechanical integrity must be preserved over time.

#### 3.3.2. Impact Resistance Comparison

**Figure 13:**
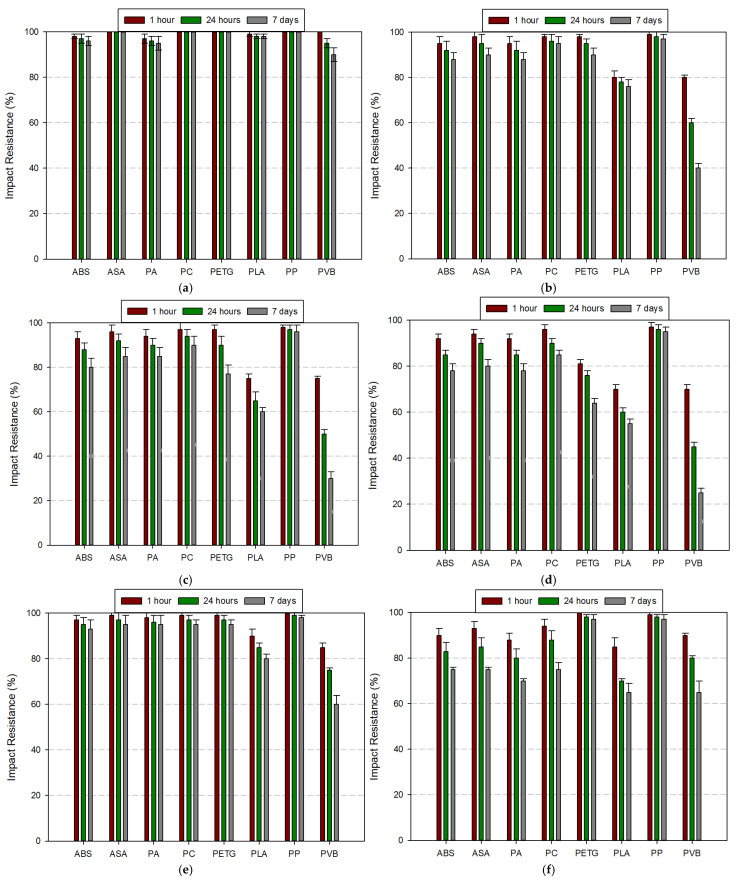

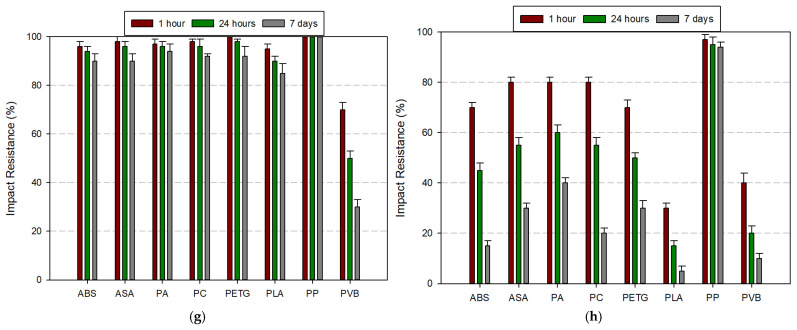
Comparison of impact resistance of filament materials according to the fluids used: (**a**) water, (**b**) ethanol (99.5%), (**c**) IPA (75%), (**d**) IPA (99%), (**e**) acetic acid (8%), (**f**) hydrochloric acid (37%), (**g**) hydrogen peroxide (30%), (**h**) acetone (99.5%).

The impact resistance of selected filament materials was evaluated under different chemical environments over three immersion durations (1 h, 24 h, and 7 days). Figure 13a–h present the normalized impact resistance values for each material and condition. The results reveal that chemical exposure significantly influences the impact performance of certain polymers over time. Acetone (99.5%) induced the most severe reduction in impact resistance, especially in PLA, PVB, PETG, and PC. PLA and PVB dropped below 20% of their original impact resistance after 7 days, whereas PETG and PC retained only about 30–50%. Conversely, PP exhibited excellent resistance, maintaining over 95%, even after prolonged exposure. ASA and PA also performed relatively well, retaining more than 60% of initial resistance. Water immersion had a negligible effect on impact resistance across all filaments. Nearly all materials maintained resistance levels above 95%, indicating high hydrolytic stability. Only PVB displayed a slight decline, suggesting some moisture sensitivity. Ethanol (99.5%) and IPA (75% and 99%) caused moderate to severe degradation in impact resistance, particularly in PLA, PVB, and PETG. PLA’s impact resistance fell by more than 50% after 7 days in all alcohol-based solvents. ABS, ASA, PA, and PP were the least affected, showing minimal changes over time. Acetic acid (8%) had selective influence depending on polymer chemistry. While ABS, ASA, and PP showed high resistance retention (>90%), PLA, PETG, and especially PVB displayed progressive degradation in impact strength. PVB dropped below 40% after one week. Hydrochloric acid (37%) caused significant reduction in impact resistance for PLA and PVB. PLA retained only ~30%, while PVB dropped further to ~25% after 7 days. PA, ASA, and PP showed remarkable stability, with less than 10% reduction. Hydrogen peroxide (30%), being a strong oxidizer, affected PLA and PVB the most. Both materials experienced more than 40% reduction over time. The remaining materials maintained acceptable performance, with PP once again demonstrating superior resistance. In summary, PVB and PLA exhibited the lowest chemical resistance in terms of impact performance across most solvents and durations. On the contrary, PP consistently retained high impact strength, followed by ASA and PA. These findings highlight the importance of selecting materials not only based on tensile strength but also considering impact resistance under relevant chemical environments.

### 3.4. Correlation Analysis Between Tensile Strength and Impact Resistance

**Figure 14:**
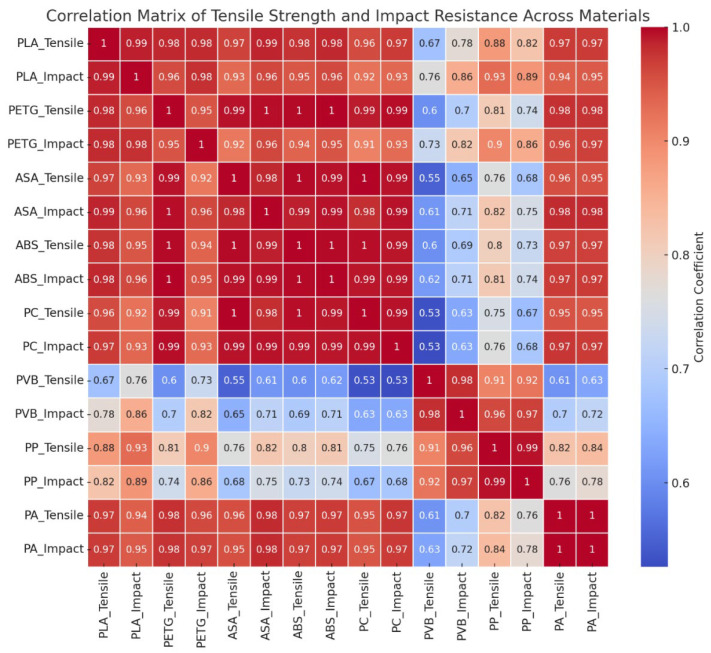
Correlation matrix illustrating the relationship between tensile strength and impact resistance for filament materials.

To explore the relationship between static (tensile) and dynamic (impact) mechanical properties under chemical stress, a correlation matrix heat map was generated (Figure 14), visualizing the Pearson correlation coefficients (r) between tensile strength and impact resistance for each material after 7 days of chemical exposure. The analysis revealed that most materials—including ABS, ASA, PETG, PA, and PLA—exhibited strong positive correlations (r ≈ 0.95–0.99). Such behavior reflects an intrinsic mechanical interdependence and provides a valuable predictive framework—changes in one property (e.g., tensile strength) may reliably indicate proportional declines in the other (e.g., impact resistance), streamlining post-exposure evaluations in applied contexts. In contrast, PVB exhibited a lower correlation coefficient (r = 0.55–0.70), implying a more decoupled mechanical response to chemical degradation. This discrepancy is likely due to PVB’s heterogeneous, highly amorphous structure and surface-dominant erosion behavior, where impact performance may degrade rapidly due to superficial brittleness, while tensile strength remains partially preserved in the core. Conversely, localized plasticization or stress-whitening may compromise tensile response without critically affecting surface impact performance. PP, despite its overall high chemical resistance, demonstrated a moderately strong correlation (r ≈ 0.85). This pattern suggests that surface-level alterations (e.g., minimal swelling, superficial crazing) might not significantly affect tensile performance but can marginally influence impact dynamics due to localized energy dissipation differences. For most FDM thermoplastics, assessing a single mechanical property post-immersion—particularly tensile strength—may serve as a proxy for total structural integrity. However, for materials with non-linear or decoupled degradation patterns (e.g., PVB), dual-property analysis is essential to avoid misleading conclusions about part viability. In chemically demanding applications, selecting materials with synchronized mechanical degradation behavior improves predictability, safety margins, and design reliability, particularly where in-service inspection and lifetime prediction are critical.

### 3.5. Scanning Electron Microscopy (SEM) Morphological Analysis

**Figure 15:**
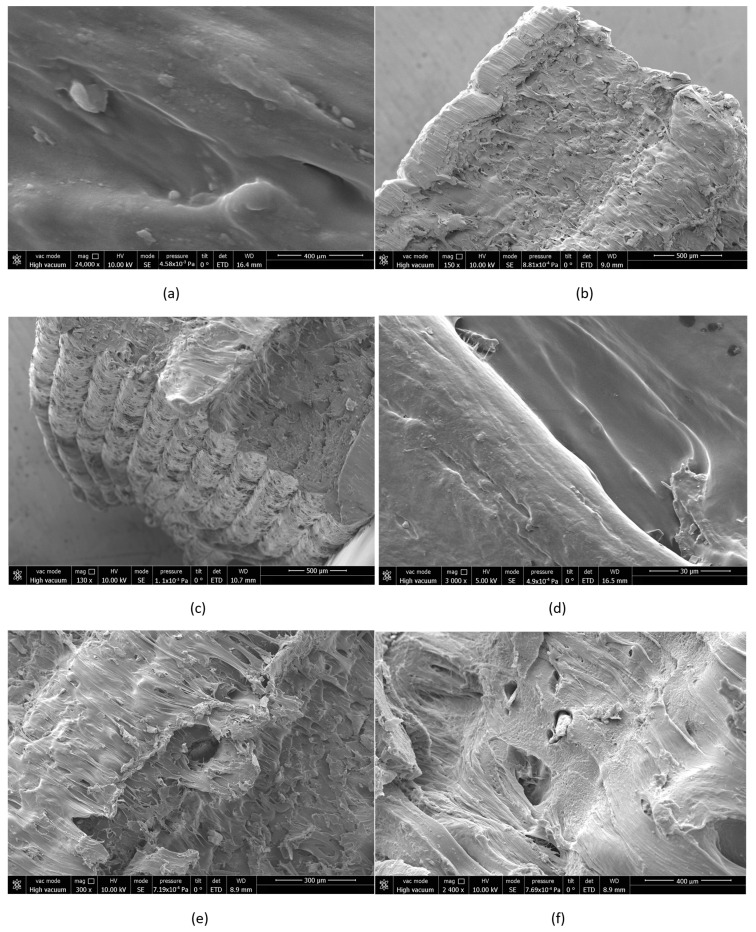
SEM micrographs of selected 3D-printed specimens before and after chemical exposure: (**a**) control PLA tensile sample with smooth surface and intact interlayer adhesion, (**b**) PLA tensile sample after 7-day acetone exposure showing brittle fracture and lamellar tearing, (**c**) PETG tensile sample exhibiting interlayer delamination following solvent immersion, (**d**) PLA tensile sample displaying surface microcracks and morphological roughening, (**e**) PETG tensile sample with signs of microvoid formation and partial disintegration, (**f**) PLA impact sample with visible chemical erosion zones and voided structures after exposure.

To further investigate the structural degradation induced by chemical exposure, SEM analysis was performed on selected tensile and impact samples. Figure 15 presents SEM images of PLA and PETG specimens before and after immersion in acetone for 7 days. In the control PLA sample (Figure 15a), a smooth surface with tightly fused layers is evident, indicating strong interlayer bonding and no significant morphological defects. However, after acetone exposure, the PLA fracture surface (Figure 15b) becomes highly irregular, showing brittle tearing and delamination, suggesting that solvent diffusion disrupted the molecular structure and weakened interlayer adhesion. The PETG sample also exhibits pronounced morphological deterioration. As shown in Figure 15c, the layered structure becomes more distinct, revealing signs of interfacial debonding. Microcracks and fractured zones (Figure 15d) were prevalent across PLA surfaces, confirming the loss of ductility and structural coherence. High-magnification images indicate the formation of microvoids and erosion zones, consistent with mechanical weakening observed in tensile (Figure 15e) and impact tests (Figure 15f). These SEM observations suggest that long-term chemical exposure leads to physical degradation mechanisms such as delamination, microcracking, and matrix erosion, especially in amorphous and semi-crystalline polymers such as PLA and PETG.

### 3.6. Summary Evaluation of Results According to Their Success Status

**Figure 16:**
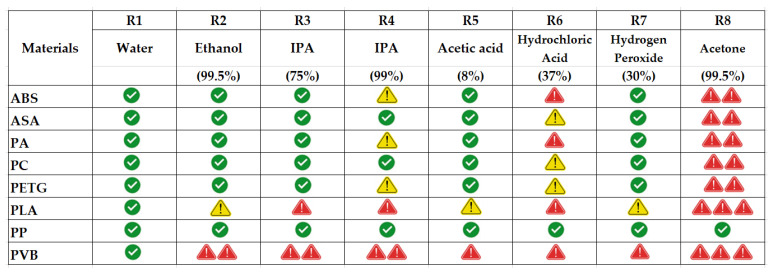
Grading the strength performance of different filaments exposed to chemicals, 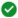: successful, 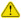: average, 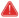: weak, 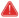

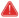
: very weak, 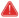

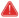

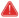
: unsuccessful.

Figure 16 provides a qualitative overview of the chemical resistance of eight commonly used FDM 3D printing filaments—ABS, ASA, PA, PC, PETG, PLA, PP, and PVB—when exposed to eight different chemical environments. Visual coding using tick green marks (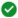), yellow caution symbols (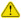), and red single (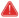) or multiple caution symbols (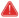

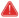
 and 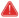

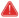

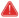
) effectively highlights the strength profile of each material. The number of red caution symbols is given in direct proportion to the losses in strength performance of the relevant filament type.

PP stands out as the most chemically stable material, maintaining resistance across all tested chemicals. This is consistent with the results of Romero et al. (2024), who emphasized PP’s semi-crystalline, non-polar structure as a key factor in limiting solvent diffusion and acid/base reactivity [52].

In contrast, PLA and PVB show the poorest resistance, especially against polar solvents (e.g., isopropyl alcohol (75% and 99%), ethanol (99.5%)) and strong acids (e.g., hydrochloric acid), represented by multiple red (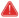

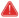
 or 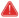

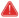

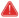
) symbols. This vulnerability is corroborated by Rezayat et al. (2024) and Csótár et al. (2025), who noted rapid loss of mechanical integrity in PLA under solvent-rich or alkaline environments [53,54].

ASA and PETG demonstrate moderate-to-high resistance in most conditions, with only minor degradation in strong solvents. Their stability has also been highlighted by Kantaros et al. (2025) and Jash et al. (2025), who attribute this to ASA’s UV-stabilized styrenic matrix and PETG’s glycol-modified backbone, which limits solvent penetration and hydrolytic breakdown [55,56].

PC and PA show selective resistance; they perform well in water, weak acids, and peroxide but degrade in concentrated organics like acetone. This selectivity aligns with structural findings by Cai et al. (2021) and Otieno et al. (2022), who reported that amorphous or hygroscopic polymers tend to suffer from environmental stress cracking or plasticization under prolonged exposure [12,22].

This visual synthesis validates the quantitative mechanical data discussed earlier and reinforces material-specific behavior in chemically aggressive environments. From an engineering perspective, PP, ASA, and PETG are recommended for solvent-prone or industrial environments, while PLA and PVB should be limited to esthetic, disposable, or chemically benign applications. Future work may focus on nanocomposite enhancements, protective coatings, or copolymer blends to extend the chemical applicability of more vulnerable materials like PLA and PVB.

## 4. Discussion

The results of this study provide a detailed and comparative assessment of the resistance of commonly used FDM 3D printing polymers to the most commonly used chemicals in the chemical industry, providing insight into how molecular structure, polarity, and crystallinity govern degradation behavior in various chemical environments. The observed degradation trends are strongly correlated with the physicochemical nature of each polymer and are in broad agreement with previously reported findings.

PP emerged as the most chemically stable filament, exhibiting less than 7% reduction in both tensile and impact properties, even after 7 days of immersion in aggressive solvents such as acetone and hydrochloric acid. These findings are consistent with the conclusions of Saidulu et al. (2022) and León-Calero et al. (2021), which attributed PP’s robustness to its saturated hydrocarbon backbone, semi-crystalline morphology, and low surface energy, all of which inhibit solvent diffusion and chemical reactivity within the polymer matrix [34,35].

At the other end of the spectrum, PLA and PVB showed extreme vulnerability, particularly under exposure to polar solvents like IPA and acetone. The severe mechanical deterioration observed in PLA confirms earlier findings by Ngo et al. (2018) and Benwood et al. (2018), which identified ester hydrolysis and amorphous chain scission as dominant degradation pathways [31,44]. For PVB, the dramatic loss in mechanical integrity aligns with Campos et al. (2022), who highlighted the role of hydrogen bonding disruption and solvent-induced swelling in accelerating failure in highly polar amorphous polymers [40].

Since PLA is the most commonly used filament material in 3D printers, the chemical degradation mechanism and reaction pathways of this material have been investigated. Hydrolysis of PLA in acidic and basic environments occurs by volume erosion and surface erosion, respectively [57]. It has been reported that the degradation of PLA occurs in both acidic and basic environments, driven by different depolymerization mechanisms. For example, chain end cleavage hydrolyzes PLA in acidic environments where protonation activates the hydroxyl group and results in the depolymerization of PLA to lactic acid [58,59].

**Figure 17:**
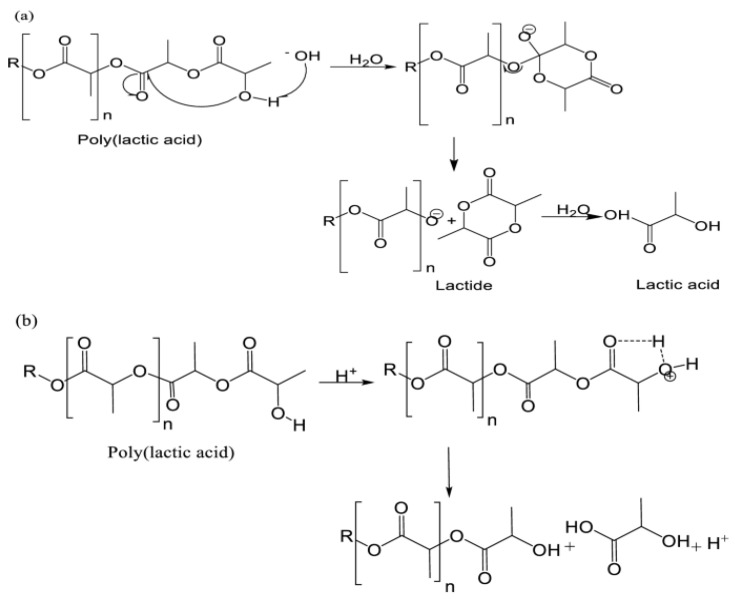
Exposure of PLA to liquid chemicals: (**a**) PLA degradation mechanism; (**b**) backbiting reaction in which PLA is depolymerized to lactide and subsequent hydrolysis occurs [60,61].

On the other hand, the backbiting reaction leads to random chain scission in basic medium, which depolymerizes PLA to lactide and subsequently hydrolyzes it [60,61], as shown in Figure 17. During hydrolysis, hydroxide ions catalyze the cleavage of the ester. At higher pH, the concentration of hydroxide ions is higher and therefore enhances the cleavage of PLA [62,63].

Polymer structures can exhibit similar chemical interactions because they contain the same types of groups that have the potential to react. In Figure 17 below, the interaction mechanism of PLA with water (Figure 17a) and acid (Figure 17b) is explained to provide general information about these pathways.

While PLA is widely recognized as a promising biodegradable polymer due to its renewability, processability, and cost-effectiveness, several intrinsic drawbacks limit its broader application in demanding environments. One of the main limitations of PLA is its brittle nature and poor impact resistance, which arises from its semi-crystalline structure and lack of chain flexibility. These characteristics make PLA susceptible to catastrophic failure under dynamic or tensile loads, especially in low-temperature or chemically aggressive conditions [64].

Additionally, PLA exhibits limited thermal stability with a relatively low glass transition temperature (~58 °C), which restricts its use in high-temperature applications such as hot-filling or microwave packaging. Moreover, PLA tends to undergo hydrolytic degradation when exposed to moisture, leading to molecular weight reduction and mechanical weakening over time [65].

From a sustainability perspective, although PLA is derived from renewable resources like corn starch and sugarcane, there are concerns regarding its competition with food sources and the environmental footprint of agricultural production [66]. Furthermore, its degradation in real environmental conditions (e.g., soil or marine environments) may be slower than expected, depending on temperature, humidity, and microbial activity [67].

Recent studies, including one by Kralin et al. (2025), have shown that blending PLA with ductile polymers, such as polybutylene adipate-*co*-terephthalate (PBAT), or using compatibilizers can enhance its mechanical and thermal behavior, but phase separation and poor interfacial adhesion often remain as major issues [68]. Therefore, while PLA offers significant advantages in terms of sustainability and printability, its inherent mechanical fragility, low thermal resistance, and slow degradation kinetics in non-industrial composting environments necessitate further modification or blending strategies for practical engineering applications.

PETG and ASA exhibited intermediate resistance, maintaining over 90% of mechanical properties in all environments except acetone. Their glycol-modified backbones (PETG) and UV-stabilized styrenic structures (ASA) confer limited polarity and moderate segmental flexibility. These observations agree with structural analyses and mechanical test results reported by Kim et al. (2021) and Ronca et al. (2022) [5,45].

ABS and PC, despite their high initial mechanical strength, demonstrated moderate chemical resistance, especially under exposure to ketones and concentrated alcohols. This behavior is congruent with studies by Stansbury and Idacavage (2016) and Otieno et al. (2022), which documented that amorphous styrenic and carbonate domains are prone to solvent-induced stress cracking and environmental embrittlement [22,28].

PA presented a chemically asymmetric profile—retaining excellent mechanical properties in water and weak acids but showing considerable loss in polar solvents and strong acids. These findings corroborate the analyses of Cai et al. (2021) and Jandyal et al. (2022), who emphasized hygroscopic swelling, plasticization, and acid-catalyzed hydrolysis as major failure modes in PA’s semi-crystalline structure [6,12].

Importantly, the correlation analysis between tensile strength and impact resistance confirms that in most polymers, both static and dynamic properties degrade concurrently under chemical attack. This mechanical coupling effect—previously described by Habiba et al. (2024) and Valls Esteve et al. (2023)—underscores the microstructural coherence of degradation mechanisms, such as interfacial weakening, crazing, and chain mobility reduction [7,11].

Overall, this study not only validates prior experimental trends in the literature but also delivers a side-by-side, time-dependent performance map for eight commercial FDM filaments under well-defined chemical exposures. The integration of tensile and impact test results across varied solvents, acids, and oxidizers, combined with multi-day immersion, provides a more application-relevant material screening framework than standard datasheet comparisons. While materials like PETG and ASA are sufficient for consumer-grade and light industrial use, only PP consistently satisfies the demands of chemically intensive environments. These insights are critical for materials engineers, product designers, and FDM practitioners seeking reliable polymer selection strategies for solvent-prone or corrosive applications.

When Figure 4, Figure 5, Figure 6, Figure 7, Figure 8, Figure 9, Figure 10 and Figure 11, which show the changes that occur as a result of chemical exposure of the substances, are examined, it is seen that the effects of the chemicals used are supportive of each other. For example, it is seen that the decrease in the strength performance of the substances treated with acetone is the greatest, while the change in the strength performance of the substances treated with water is the least and gives similar results in each test. This is thought to be due to the chemical structure of the water molecule having a less destructive/reactive effect on the polymer structure compared to both acids and alcohols.

In addition, it is possible to see that the concentration of the chemicals used is an important factor in deterioration when the data in Figure 4, Figure 5, Figure 6, Figure 7, Figure 8, Figure 9, Figure 10 and Figure 11 is examined. For example, it is seen that the deterioration of the higher (IPA 99%) concentration solution with the polymer is greater than the more dilute solution (IPA 75%). Again, when the data is examined, it is seen that acetic acid, which is a weak acid, causes less deterioration than hydrochloric acid, which is a strong acid. This is an expected result because the higher the ionizability, the more interaction it will have with the polymer. The obtained data supports this.

## 5. Conclusions

This study systematically investigated the chemical resistance profiles of eight commercially available FDM 3D printing materials—ABS, ASA, PA, PC, PETG, PLA, PP, and PVB—subjected to prolonged immersion in industrially relevant chemicals, including water, ethanol (99.5%), IPA (75% and 99%), acetic acid (8%), hydrochloric acid (37%), hydrogen peroxide (30%), and acetone (99.5%). Strength performances were quantified through tensile strength and Izod impact resistance testing at 1 h, 24 h, and 7-day exposure intervals.

The results revealed distinct, material-specific degradation patterns that closely correspond to molecular structure, chemical polarity, and crystallinity. PP exhibited the highest chemical durability, retaining 93–100% of its original mechanical properties across all tested environments. In contrast, PLA and PVB experienced severe degradation in solvent-rich media, especially 99% IPA and acetone (99.5%), where tensile strength dropped below 30% and impact resistance fell below 10% after 7 days.

- PP is highly recommended for harsh chemical environments involving strong acids, oxidizers, or organic solvents, making it ideal for laboratory apparatus, fluid handling systems, and chemical-resistant mechanical enclosures;
- PETG and ASA, which maintained over 90% mechanical integrity in most conditions except acetone (99.5%), are suitable for consumer products, lightweight structural components, and UV-exposed outdoor applications;
- PLA and PVB, while cost-effective and esthetically advantageous, are best reserved for non-load-bearing and chemically benign contexts, such as educational prototypes, display parts, or biodegradable packaging;
- ABS, PC, and PA demonstrated moderate chemical resistance, and their use in solvent-prone or acidic environments should be approached with engineering caution and material-specific testing.

The correlation analysis between tensile strength and impact resistance validated a strong mechanical coupling (r ≥ 0.95) in most materials, enabling the use of one metric as a predictive indicator for the other. However, materials like PVB, which showed lower inter-property correlation (r < 0.7), necessitate separate mechanical evaluations for comprehensive durability assessment.

By integrating chemical immersion testing, mechanical performance analysis, and correlation-based property modeling, this study offers a data-driven material selection framework tailored to real-world, chemically aggressive conditions. These insights transcend conventional datasheet specifications, equipping engineers and designers with a pragmatic, application-centered toolset for optimizing material choice in the expanding domain of additive manufacturing under chemical stress.

As a result of the experimental studies, both the concentration of the chemicals used and the strength of the acid bases used were found to be an effective factor in the degradation of polymers. It can also be said that the most polymer-degrading chemical is acetone (99.5%), while the least effective structure is water.

## Figures and Tables

**Table 1:** Trade names of filament materials, manufacturer, and country of origin.

Materials	Trade Names	Manufacturers	Countrys of Origin
ABS	ABS-M30	Stratasys	Minnesota, USA
ASA	ASA-Pro	Polymaker	Houston, USA
PA	Nylon 230	Taulman 3D	California, USA
PC	PC-Max	Polymaker	Houston, USA
PETG	PETG-X	Prusa	Prague, Czech Republic
PLA	PLA+ Premium	eSUN	Shenzhen, China
PP	PP-Natural	Ultimaker	Geldermalsen, Netherlands
PVB	BVOH Blend	Fillamentum	Hulin, Czech Republic

**Table 2:** Material type, properties and common application areas of filaments.

Material	Properties	Applications
ABS	High impact resistance, high heat resistance.	Automotive parts, electronic housings, toys.
ASA	UV and weather-resistant, good heat resistance.	Outdoor furniture, automotive parts, garden equipment.
PA	Flexible, high wear resistance, strong mechanical properties.	Gears, bearings, textile applications.
PC	High durability, impact-resistant, heat-resistant.	Safety glasses, electronic housings, machinery parts.
PETG	Strong, flexible, resistant to water and chemicals.	Durable prototypes, food packaging, chemical-resistant.
PLA	Biodegradable, derived from renewable resources.	Prototyping, educational prints, temporary projects.
PP	Chemical resistance, flexibility, low density.	Chemical containers, automotive parts, food packaging.
PVB	Transparency, smooth finish, good adhesion properties.	Laminated glass layers, decorative parts, adhesive layers.

**Table 3:** Chemicals used in the study: formulas, codes, properties, and applications.

Chemicals	Formulas	Codes	Properties	Applications
Water	H_2_O	R1	Baseline, neutral substance	Testing material reactions, general-purpose solvent
Ethanol (99.5%)	C_2_H_6_O	R2	Alcohol, solvent, disinfectant	Surface cleaning, hand sanitizers, alcoholic beverages
Isopropyl Alcohol (IPA) (75%)	C_3_H_8_O	R3	Solvent, cleaning and sterilization agent	Cleaning, sterilizing surfaces, electronics
Isopropyl Alcohol (IPA) (99%)	C_3_H_8_O	R4	Solvent, cleaning and sterilization agent	Cleaning, sterilizing surfaces, electronics
Acetic Acid (8%)	CH_3_COOH	R5	Weak acid, antibacterial properties	Household cleaning, food preparation (vinegar)
Hydrochloric Acid (37%)	HCl	R6	Strong acid, highly reactive	Industrial cleaning, chemical processing
Hydrogen Peroxide (30%)	H_2_O_2_	R7	Strong oxidizer, sterilizing agent	Medical sterilization, cleaning, bleaching
Acetone (99.5%)	C_3_H_6_O	R8	Strong solvent, dissolves many plastics	Surface cleaning, paint thinning, nail polish remover

**Table 4:** Technical parameters and features of Creality K1 max 3D printer.

Parameter	Value/Description
Layer Height	0.20 mm
Infill Density	100%
Printing Speed	50 mm/s
Build Orientation	Vertical (*Z*-axis)
Nozzle Temperature	PLA: 210–215 °C ABS/ASA: 260–270 °C PETG: 220–250 °C PA: 240–270 °C PP: 230–260 °C PVB: 210–240 °C PC: 260–310 °C
Bed Temperature	PLA: 60 °C ABS/ASA: 110 °C PETG: 50-80 °C PA: 50 °C PP: 65–85 °C PVB: 40–65 °C PC: 90–120 °C
Filament Storage	Stored in a desiccated cabinet with humidity maintained below 15%

**Table 5:** Control tensile strength and Izod impact test results and ±SD of the experimental samples obtained before exposure to liquid chemicals.

Materials	Tensile Strength (MPa) Value ± SD	Izod Impact Test (J/m) Value ± SD
ABS	35.03 ± 2.21	200.51 ± 5.37
ASA	43.15 ± 1.42	145.26 ± 3.07
PA	60.23 ± 1.17	84.12 ± 1.09
PC	62.09 ± 3.37	725.06 ± 8.46
PETG	40.31 ± 1.08	75.77 ± 1.34
PLA	54.28 ± 1.75	16.36 ± 1.25
PP	33.11 ± 2.05	150.51 ± 4.79
PVB	27.04 ± 1.34	30.06 ± 2.03

## Data Availability

Data are contained within the article. Further inquiries can be directed to the author.
